# RE: Reactive macrophage activation syndrome (MAS) in a patient with parvovirus B19 infection, lymphocytic lichenoid vasculitis, urticaria and angioedema

**DOI:** 10.4103/0256-4947.62827

**Published:** 2010

**Authors:** Luis Gonzalez-Granado

**To the Editor:** I read with keen interest the article published by Soldo-Juresa et al in the last issue of the journal.^1^ I would like to make some comments: First, Still disease should be kept in mind whenever macrophage activation syndrome is considered in a patient with the clinical picture shown. In fact the patient's condition fulfills the criteria published by Yamagushi for Still disease.^2^ I would like to remark that the measurement of soluble IL-2R (also known as CD25s) could be helpful in distinguishing between Still disease and MAS.^3^ Furthermore, the development of MAS in Still disease is not infrequent and intravenous gamma globulins have shown promising results in treatment.^4^,^5^ Second, as the authors rule out a C1q-linked vasculitis, C4 and alternative complement pathway activity should be measured. It is well known that in esterase inhibitor type II levels of C1-inhibitor esterase are normal although C4 is decreased due to low functional C1-inhibitor levels.^6^ Hypocomplementemic vasculitis has been also described in patients with factor I deficiency.^7^ I have attended recently a patient with vasculitis secondary to factor I deficiency. This factor was not measured by the author in this patient. Third, the authors recognize that DNA of parvovirus B-19 can persist in bone marrow for years. This is why we should distinguish between MAS secondary to viral infection or underlying Still disease that needs long-term treatment and follow-up.
